# Molecular Insights into Human Brain Evolution

**DOI:** 10.1371/journal.pbio.0030050

**Published:** 2005-03-15

**Authors:** Jane Bradbury

## Abstract

As a species, we pride ourselves on the uniqueness of our brain. But comparisons with other species may tell us how our unique brains evolved

As a species, we pride ourselves on the uniqueness of our brain. Relative to our body size, the human brain is bigger than that of any other animal. It may also contain unique structures and patterns of organisation that presumably underlie our intelligence and ability to manipulate our environment. But how did our unique brain originate, and under what selective pressures did it evolve? Some of the answers may lie in the genetic differences that researchers are now uncovering between us and our closest relatives.

## What Is So Different about the Human Brain?

**Figure 1:**
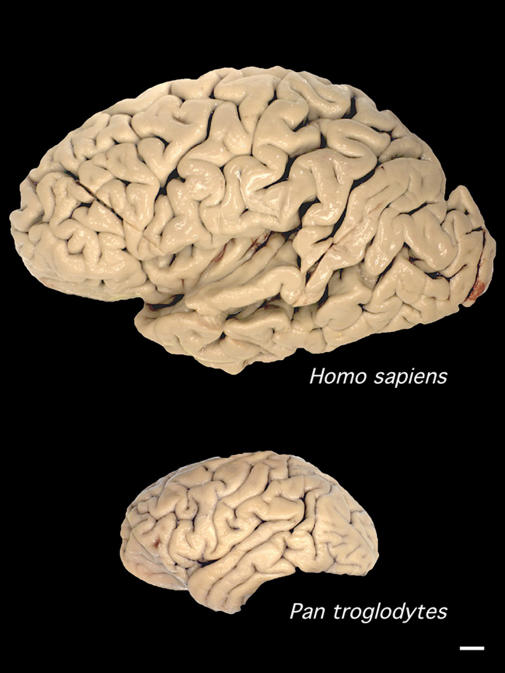
Comparison of a Human and a Chimpanzee Brain Scale bar = 1 cm (Image: Todd Preuss, Yerkes Primate Research Center)

When we compare our brain to those of other animals, the first thing that strikes us is its size. Human brains weigh on average 1,300 grams; a squirrel brain weighs six grams. Some of this difference is because, as larger animals, we need more brain to run our bodies. However, the brains of our nearest relatives, the great apes, weigh only 300–500 grams, even though their body size is similar to ours (Figure 1). “Humans sit on the top of the pile when it comes to relative brain size”, notes geneticist Bruce Lahn (University of Chicago, Illinois, United States) (see Box 1).

**Box 1. Nothing like a Whale**

Just how unique is human brain evolution? Neuroscientist Lori Marino (Emory University, Atlanta, Georgia, United States) and her colleagues have used computed tomography to estimate the body and brain size of 36 fossil whale species and have compared these data with those for modern toothed whales. Relative to body size, whales and dolphins have the next biggest brains to us, bigger even than chimpanzees, and, says Marino, “there have been dolphins swimming in the oceans with huge brains for more than 15 million years. We are really the new kids on the block.”

Like in humans and other primates, the neocortex in whale brains is huge, but its structure is very different to that of our neocortex. Whales have been independent of other lineages for about 60 million years, notes Marino, and haven't shared a common ancestor with primates for 94 million years. “Nevertheless, during evolution, whales have converged upon very similar capacities and behaviours to those of primates, including a highly developed social structure, which tells us that there is more than one way to evolve a complex intelligence.”

Throughout mammalian and primate evolution, there has been a gradual increase in brain size, superimposed with “spikes” of fast growth such as the tripling in human brain size that occurred about 1.5 million years ago, 4 million years after the human lineage diverged from that of the great apes. “Even in the ape lineage, the brain has been expanding but along the human lineage it has really taken off”, says Lahn.

In addition, over time, different parts of our brain have increased in size at different rates. The cerebral cortex has expanded more than other areas, and within the cortex, some areas have expanded differentially while others have lagged behind.

“Humans sit on the top of the pile when it comes to relative brain size.”

Paleoanthropologist Ralph Holloway (Columbia University, New York, United States) uses endocasts to look for macroscopic differences in the brains of our human ancestors. “We fill human fossil skulls with vulcanised rubber and once it has set, we pull it out of the large hole at the base of the skull and the rubber snaps back into the shape of the skull”, Holloway explains. Endocasts are particularly useful for comparing brain sizes, but they also provide information on when the asymmetries that are present in our brain first appeared. These often reflect cerebral specialisation, and Holloway believes that some of the asymmetries he sees in human fossil skulls may indicate when our ancestors acquired language.

**Figure 2:**
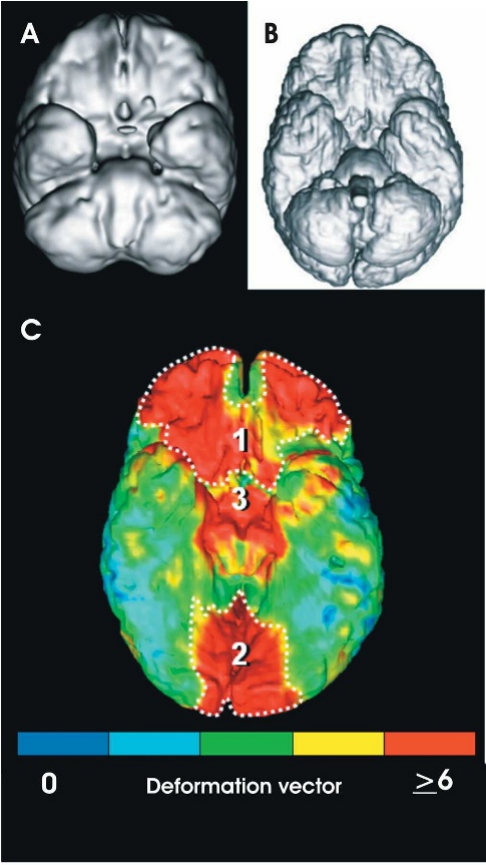
Magnetic Resonance Imaging of Brains Three-dimensional reconstruction of a reference bonobo (pygmy chimpanzee) brain (A) and a reference human brain (B) after magnetic resonance imaging and normalisation of absolute brain sizes. The virtual bonobo brain has been transformed into the virtual human brain using an elastic deformation algorithm. The local deformation vectors are colour-coded and projected onto the virtual human brain (C). The most dramatic changes in brain shape occur in (1) the ventro-orbital prefrontal cortex, (2) the ventral stream of the visual cortex, and (3) the hypothalamic neuroendocrine region. (Image: Karl Zilles, Hartmut Mohlberg, and Peter Pieperhoff, Research Center Jülich)

More details about how the shape of our brain differs from that of our closest living relatives are emerging from the work of neuroscientist Karl Zilles (Institute of Medicine, Research Center Jülich, Germany). He prepares magnetic resonance images of monkey, ape, and human brains and then uses a nonlinear elastic algorithm to transform one brain into another (Figure 2). “We know what forces we have to apply to the images to do this”, he explains, “which tells us which areas of the brain have changed most during primate evolution”. Zilles and his colleagues also are currently using molecular imaging techniques to update the existing maps of the different areas within our brains. Until we have this information, it is hard to make meaningful comparisons between our brain and that of chimpanzees. Already, Zilles has discovered that there is much more interindividual variation in human brain organisation than anyone suspected. This means, says Zilles, “that a general statement like ‘the neocortex is bigger in human brains than in ape brains’ actually tells us very little. It gives us the general direction that evolution has taken but not whether an ape brain is different because of its sensory, motor, or association areas.”

Scientists are also using other techniques to investigate more subtle changes in the organisation of the human brain compared to the brains of other mammals and primates. Indeed, says Holloway, the reorganisation of the brain during evolution has been at least important as its increase in size. Neurobiologist John Allman (California Institute of Technology, Pasadena, California, United States) and his collaborators, for instance, have discovered that a special type of large spindle-shaped neuron, first described in the early 20th century by Constantin von Economo, is unique to apes and humans and much more numerous in the latter. These neurons are found in brain areas that are implicated in decision making in uncertain situations so, Allman speculates, they may help humans to interact rapidly in complex social situations.

## Costs and Benefits

A bigger, more complex brain may have advantages over a small brain in terms of computing power, but brain expansion has costs. For one thing, a big brain is a metabolic drain on our bodies. Indeed, some people argue that, because the brain is one of the most metabolically expensive tissues in our body, our brains could only have expanded in response to an improved diet. Another cost that goes along with a big brain is the need to reorganise its wiring. “As brain size increases, several problems are created”, explains systems neurobiologist Jon Kaas (Vanderbilt University, Nashville, Tennessee, United States). “The most serious is the increased time it takes to get information from one place to another.” One solution is to make the axons of the neurons bigger but this increases brain size again and the problem escalates. Another solution is to do things locally: only connect those parts of the brain that have to be connected, and avoid the need for communication between hemispheres by making different sides of the brain do different things. A big brain can also be made more efficient by organising it into more subdivisions, “rather like splitting a company into departments”, says Kaas. Overall, he concludes, because a bigger brain *per se* would not work, brain reorganisation and size increase probably occurred in parallel during human brain evolution. The end result is that the human brain is not just a scaled-up version of a mammal brain or even of an ape brain.

For natural selection to work, the costs of brain evolution must be outweighed by the advantages gained in terms of fitness. For many years, explains ecological psychologist Robin Dunbar (University of Liverpool, United Kingdom), “people thought that the ability to hunt or forage better was what drove the evolution of our brains. But a better diet had to come before we could grow a bigger brain.” Dunbar believes instead that brain evolution in primates and more generally in mammals “has been driven by the need to manage social relationships, and in primates, in particular, to coordinate coherence in social groups through time and space”. More complex social interactions, he says, mean that individuals are better able to pool resources to solve problems like finding food, and so they survive better.

This theory, says Dunbar, is supported by a correlation between social group size and neocortex size across primates and modern humans. Furthermore, during primate brain evolution, the trend has been to add more material to the front than the back of the brain. The front of the brain is where information from the rest of the brain is interpreted, and the capacity to interpret information underlies social interactions, says Dunbar. The number of problem-solving cognitive tasks you can do may well depend on how much frontal lobe volume you have and how it is organised. Just think of how few moves you can run a chess game into the future with a 1980s personal computer compared to a 21st century mainframe machine, he suggests.

The human brain is not just a scaled-up version of a mammal brain or even of an ape brain.

## The Genetics of Human Brain Evolution

**Figure 3:**
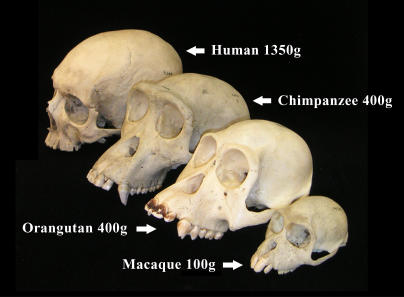
Primate Brain Sizes These skulls are from the Harvard Museum of Comparative Zoology. (Image: Christopher Walsh, Harvard Medical School)

Selective pressures like those considered by Dunbar and, before him, by scientists like Holloway work on the raw material of random gene mutations, and molecular biologists now have some clues to the gene changes that may underlie brain evolution. Take brain size, for example (Figure 3). Research groups, including those led by Lahn, neurologist Christopher Walsh (Harvard Medical School, Boston, Massachusetts, United States), and clinical geneticist Geoffrey Woods (University of Leeds, United Kingdom), wondered whether the genes that cause microcephaly, an inherited human disorder in which brain size is greatly reduced, might include genes involved in human brain evolution. In 2002, mutations in the genes *ASPM (abnormal spindle-like microcephaly associated)* and *microcephalin* were identified as two causes of microcephaly. Three groups have since reported that both these genes have been under selective pressure during primate evolution. *ASPM* encodes a protein involved in spindle formation, so it is tempting to think that changes in its sequence might result in an increased rate of cell division and hence brain size. But, cautions Walsh, “we really have no idea yet how or even if *ASPM* is involved in brain evolution”.

Both Lahn and Walsh believe that *ASPM* and *microcephalin* may be only the tip of the iceberg when it comes to genes that have helped to shape our brains. For example, Walsh has recently reported that deletion of a gene called *Nde1* produces mice with very small brains. “Our experiments indicate that the loss of *Nde1* causes neurons to mature prematurely. This stops them dividing so the mice end up with small brains”, explains Walsh, who is now investigating whether human *NDE1* variants have been positively selected during human evolution.

Lahn is also searching for additional candidate genes that might help to explain how our brains evolved. In a recent *Cell* paper, Lahn and his colleagues identify several hundred genes that are involved in nervous system biology and show that, as a group, there are significantly higher rates of protein evolution in these genes in primates than in rodents. Protein evolution rates are particularly high in the lineage leading from ancestral primates to humans, notes Lahn, “so some of these genes may regulate brain size and behaviour”. However, he warns, as with *ASPM* and *microcephalin*, “definitive proof for this will only come from functional studies, which are difficult to do”.

## Enter Glutamate Dehydrogenase

For one gene, evidence for an effect on brain function may be emerging. Geneticist Henrik Kaessmann (University of Lausanne, Switzerland) studies the origin of new genes in primates, in particular genes that arise when a DNA copy of an mRNA transcribed from an existing gene is integrated back into the genome. Usually this new “retrocopy” is not expressed, but if the DNA inserts near an active promoter, it can become a transcribable “retrogene”. This is the origin of *GLUD2*, a retrogene derived from *GLUD1*, which encodes glutamate dehydrogenase. *GLUD2*, which first appeared 18–23 million years ago in hominoids, probably immediately picked up a brain-specific promoter and then over the next few million years acquired two critical amino acid changes, explains Kaessmann. These allow *GLUD2*-encoded glutamate dehydrogenase to work better in the brain than the *GLUD1*-encoded enzyme. Because glutamate dehydrogenase recycles the neurotransmitter glutamate, the presence of *GLUD2* may permit a higher neurotransmitter turnover and greater neuronal activity in hominoid brains than is possible in monkey brains, which lack *GLUD2*, suggests Kaessmann.

## Gene Expression

Kaessmann plans to search his extensive database of retrocopies in the human genome for other functional genes that could, like *GLUD2*, be implicated in brain evolution. By contrast, evolutionary neurobiologist Todd Preuss (Yerkes Primate Research Center, Emory University, Atlanta, Georgia, United States) hopes to identify genes involved in human brain evolution by comparing gene expression patterns in different primates. Preuss, who began training as a paleoanthropologist before turning to neuroscience, has been comparing post-mortem human and chimpanzee brains since the mid 1990s, believing that “if we want to understand human brain evolution, we really have to compare humans with chimpanzees, our nearest relatives”, even though chimp brains have been evolving separately from ours for 5–7 million years. But, warns Preuss, “we have to do these studies now. There are few chimps left and if we lose the opportunity to study them and their brains, we will lose forever a fundamental source of insight into our own species.”

To begin with, Preuss used staining techniques that exploit antibodies to examine the neural components of chimpanzee and human brains. Then in 1998, he was asked to collaborate in a microarray project. “My antibody approach was very labour intensive so I jumped at the opportunity to screen 10,000 genes at once”, he says.

Preuss and his collaborators now know that more than 100 genes are differentially expressed in chimpanzee and human brains. “Importantly, when we go back into tissue with probes for these gene products, in some cases there are remarkably different spatial patterns of expression in humans, chimps, and macaques”, notes Preuss. “We don't know yet what these differences mean in terms of functional organisation in these different brains but our results open up new and exciting vistas”, particularly since many of the differentially expressed genes have not previously been considered as being potentially involved in brain evolution. The microarray data produced by Preuss and other researchers also indicate that many of the gene expression changes that have occurred during brain evolution involve gene upregulation. For example, there is increased expression of genes involved in metabolism, synaptic organisation, and synaptic function. “All told, it seems that the human brain may be more dynamic than ape or monkey brains”, says Preuss. “The human brain seems to be running hot in all sorts of ways.”

## Scratching at the Surface

As far as understanding how our brains evolved, more questions remain than have been answered. One problem is that we don't really know enough about how our brains differ from those of other mammals and primates, although work by Zilles and others is helping here. We also know very little about how the areas of our brain are physically linked up, and we need to understand that before we can see how we differ from our nearest relatives. And as far as identifying the gene changes that were selected during evolution, although we have several candidates, we don't know how or if these gene variants affect our cognitive abilities. It is one thing, concludes Dunbar, to identify genetic or anatomic differences between human and ape brains, but quite another to know what they mean in terms of actual cognitive processes.

## References

[pbio-0030050-b1] Burki F, Kaessmann H (2004). Birth and adaptive evolution of a hominoid gene that supports high neurotransmitter flux. Nat Genet.

[pbio-0030050-b2] Dorus S, Vallender EJ, Evans PD, Anderson JR, Gilbert SL (2004). Accelerated evolution of nervous system genes in the origin of Homo sapiens. Cell.

[pbio-0030050-b3] Dunbar RIM (2003). The social brain: Mind, language, and society in evolutionary perspective. Annu Rev Anthropol.

[pbio-0030050-b4] Feng Y, Walsh CA (2004). Mitotic spindle regulation by *Nde1* controls cerebral cortex size. Neuron.

[pbio-0030050-b5] Holloway RL, Broadfield DC, Yuan MS, Schwartz JH, Tattersall I (2004). Brain endocasts—The paleoneurological evidence. The human fossil record.

[pbio-0030050-b6] Kaas JH (2004). Evolution of somatosensory and motor cortex in primates. Anat Rec.

[pbio-0030050-b7] Marino L, McShea DW, Uhen MD (2004). Origin and evolution of large brains in toothed whales. Anat Rec.

[pbio-0030050-b8] Preuss TM, Gazzaniga MS (2004). What is it like to be a human?. The cognitive neurosciences III, 3rd ed.

[pbio-0030050-b9] Preuss TM, Cáceres M, Oldham MC, Geschwind DH (2004). Human brain evolution: Insights from microarrays. Nat Rev Genet.

[pbio-0030050-b10] Vallender EJ, Lahn BT (2004). Positive selection on the human genome. Hum Mol Genet.

